# Supplementary material for: A systematic analysis of genetic interactions and their underlying biology in childhood cancer
Source: Commun Biol. 2021 Oct 6;4:1139. doi: 10.1038/s42003-021-02647-4 (PMC8494736; doi:10.1038/s42003-021-02647-4)

A systematic analysis of genetic interactions and their underlying biology in childhood cancer

Josephine T. Daub, Saman Amini, Denise J.E. Kersjes, Xiaotu Ma, Natalie Jäger, Jinghui Zhang, Stefan M. Pfister, Frank C.P. Holstege, Patrick Kemmeren

Supplementary information

Supplementary Table 1: Number of tumors and samples removed during filtering

DKFZ	Total		WGS		WES	
	Tumors	Samples	Tumors	Samples	Tumors	Samples
**In downloaded files**	**914**	**961**	**542**	**547**	**372**	**414**
Hypermutators	7	7	5	5	2	2
Single end sequencing samples	10	10	0	0	10	10
Relapses	35	82	2	7	33	75
**Remaining after sample filtering**	**862**	**862**	**535**	**535**	**327**	**327**
Samples without functional mutations	33	33	12	12	21	21
**Remaining after mutation filtering**	**829**	**829**	**523**	**523**	**306**	**306**
**TARGET**	**Total**		**WGS**		**WES**	
	Tumors	Samples	Tumors	Samples	Tumors	Samples
**In downloaded files**	**1,648**	**1,672**	**653**	**655**	**1,115**	**1,115**
Missing metadata	3	3	1	1	2	2
Hypermutators	2	2	0	0	2	2
**Remaining after sample filtering**	**1,643**	**1,667**	**652**	**654**	**1,111**	**1,111**
Samples without functional mutations	11	11	5	5	6	6
**Remaining after mutation filtering**	**1,632**	**1,656**	**647**	**649**	**1,105**	**1,105**

Note: after filtering one tumor per patient remains, so final tumor counts can be read as final patient counts.

Supplementary Table 2: MLA scores of candidate gene pairs

MLA scores for genes in candidate genetic interactions and absolute difference between their MLA scores (ΔMLA). 'Suspect' gene pairs are marked in grey and have at least one gene with MLA > 3 and have ΔMLA > 3 in case of mutual exclusivity or both MLA > 3 in co-occurring gene pairs. For the mutated genes involved in suspect gene pairs, the cancer sub type in which they are enriched are listed in parentheses. The table is sorted on ΔMLA for each data set.

data set	cancer type	gene-1 (subtype)	gene-2 (subtype)	MLA-1	MLA-2	ΔMLA	suspect
**TARGET**	T-ALL	NOTCH1	USP7 (TAL1)	4.7	-1.6	6.4	x
	T-ALL	JAK3 (HOXA)	PTEN (TAL1)	4.3	-1.7	6.0	x
	T-ALL	PHF6 (TLX3)	PTEN (TAL1)	3.5	-1.7	5.2	x
	T-ALL	PHF6 (TLX3)	USP7 (TAL1)	3.5	-1.6	5.1	x
	T-ALL	DNM2	USP7	2.9	-1.6	4.5	-
	T-ALL	MAGI1	NOTCH1	0.3	4.7	4.4	x
	T-ALL	PTEN	WT1	-1.7	2.5	4.3	-
	T-ALL	USP7	WT1	-1.6	2.5	4.2	-
	B-ALL	KRAS	TP53	3.0	-0.8	3.8	-
	T-ALL	FBXW7	JAK3 (HOXA)	1.1	4.3	3.2	x
	T-ALL	FBXW7	PTEN	1.1	-1.7	2.8	-
	WT	DROSHA	TP53	-1.1	1.7	2.8	-
	T-ALL	JAK3	STAT5B	4.3	1.7	2.6	-
	T-ALL	PHF6	PIK3R1	3.5	1.0	2.5	-
	AML	NRAS	WT1	2.7	0.5	2.1	-
	AML	KIT	NRAS	0.9	2.7	1.8	-
	B-ALL	PXDN	ZNF582	4.1	2.8	1.3	-
	B-ALL	CRLF2	NRAS	0.3	1.5	1.3	-
	AML	CEBPA	CSF3R	0.1	1.3	1.1	-
	B-ALL	FLT3	KRAS	2.0	3.0	1.0	-
	T-ALL	JAK1 (HOXA)	JAK3 (HOXA)	3.5	4.3	0.8	x
	T-ALL	NRAS	WT1	3.3	2.5	0.8	-
	AML	FLT3	IDH2	0.4	1.1	0.7	-
	AML	FLT3	KIT	0.4	0.9	0.5	-
	AML	CEBPA	WT1	0.1	0.5	0.4	-
	AML	IDH2	NPM1	1.1	0.7	0.4	-
	AML	KIT	WT1	0.9	0.5	0.4	-
	WT	CTNNB1	EFCAB6	-0.6	-0.8	0.2	-
**DKFZ**	HGG-K27M	ACVR1	TP53	-1.2	2.1	3.3	-
	HGG-K27M	ACVR1	H3F3A	-1.2	1.4	2.6	-
	HGG-K27M	H3F3A	HIST1H3B	1.4	-1.1	2.5	-
	HGG-other	ATRX	H3F3A	0.7	1.9	1.2	-
	MB-SHH	PTCH1	SMO	-0.8	0.2	0.9	-
	MB-WNT	DDX3X	KMT2D	0.7	1.2	0.5	-
	MB-SHH	KMT2D	PTCH1	-0.9	-0.8	0.1	-
	HGG-K27M	ACVR1	HIST1H3B	-1.2	-1.1	0.1	-

Supplementary Note 1: Literature review of candidate genetic interactions and their underlying explanations

DKFZ

<table>
<tr><th>cancer type</th><th>gene1</th><th>gene2</th><th>co/me</th><th>possible explanation(s)</th><th>reference(s)</th></tr>
<tr><td>HGG-K27M</td><td>ACVR1</td><td>HIST1H3B</td><td>co*</td><td>cooperation</td><td>[52]</td></tr>
<tr><td></td><td colspan="5">Cooperation: Mutated Acvr1 arrests differentiation of oligodendroglial lineage cells and cooperates with Hist1h3b^{K27M} and mutated Pik3ca to generate high-grade diffuse gliomas. Acvr1 upregulates transcription factors which control differentiation and DIPG cell fitness [52].</td></tr>
<tr><td></td><td>ACVR1
ACVR1
H3F3A</td><td>H3F3A
TP53
HIST1H3B</td><td>me*
me*
me*</td><td>subtype</td><td>[32,33]</td></tr>
<tr><td></td><td colspan="5">Subtype: Patients in the HGG-K27M group can be divided in two sub-groups, defined by K27M mutations in either the HIST1H3B or H3F3A genes that code for histones H3.1 and H3.3 respectively. Typically, patients in the H3.1 subgroup also carry ACVR1 mutations (see above), and patients in the H3.3 subgroup are enriched for TP53 mutations (see below), explaining the ACVR1-H3F3A, ACVR1-TP53 and HIST1H3B-TP53 (PAN) mutual exclusivity relationships found in our study.</td></tr>
<tr><td>(PAN)</td><td>HIST1H3B</td><td>TP53</td><td>me</td><td>subtype</td><td>[32,33]</td></tr>
<tr><td></td><td colspan="5">Subtype: TP53 mutations are enriched in the H3.3 sub-group (although not significantly in our test) resulting in mutual exclusivity between TP53 and H3.1</td></tr>
<tr><td>HGG-other</td><td>ATRX</td><td>H3F3A</td><td>co*</td><td>cooperation</td><td>[39,53,54]</td></tr>
<tr><td></td><td colspan="5">Cooperation: The H3F3A mutations in the HGG-other group are not K27M, but G34R/V H3 mutations, another recurrent H3F3A alteration in high grade gliomas. ATRX regulates chromatin remodeling and transcription and plays an important role in maintaining genome stability by recruiting H3.3 at telomeres and pericentric heterochromatin. It has been suggested that the loss of ATRX could prevent mutant H3.3 from altering transcription of specific oncogenes[39], which would suggest a co-operative role of both altered genes in tumorigenesis [53,54]. The co-occurring relationship between H3F3A and ATRX is also found between other H3F3A mutations, such as K27M, and the detection of this candidate pair in the PAN cancer test could therefore point to a signal in both HGG subgroups.</td></tr>
<tr><td>MB-SHH</td><td>PTCH1</td><td>SMO</td><td>me</td><td>pathway epistasis</td><td>[36]</td></tr>
<tr><td></td><td colspan="5">Pathway epistasis: SMO and PTCH1 are both part of the Sonic Hedgehog (Shh) pathway, which is assumed to have a tumor driver role in MB-SHH. In this pathway, PTCH1 normally inhibits activation of SMO, but the presence of Hedgehog (Shh) leads to suppression of PTCH1 and consecutive activation of the Shh pathway by SMO. It has been shown that SMO inhibition is most effective in MB-SHH patients with SMO or PTCH1 mutations [36], suggesting that SMO mutations found in MB-SHH samples do not have a knock-out effect of the gene, but more likely release or circumvent its inhibition by PTCH1. This might explain the ME relationship found between altered PTCH1 and SMO genes, as both have the same effect and therefore are redundant mutations.</td></tr>
<tr><td></td><td>KMT2D</td><td>PTCH1</td><td>me</td><td>pathway epistasis</td><td>[55,56]</td></tr>
<tr><td></td><td colspan="5">Pathway epistasis: KMT2D (also known as MLL2) is a H3K4 methyltransferase [55] and it plays a role in an epigenetic switch induced by Shh signaling that activates target genes -such as PTCH1- by removing H3K27me3 and increasing levels of H3K4me3 [56]. Mutations in KMT2D could disrupt H3K4me3 regulation and as a result inactivate downstream genes such as PTCH1 in the Shh pathway, which would have the same effect as PTCH1 inactivating mutations, explaining the mutual exclusive relation between KMT2D and PTCH1.</td></tr>
<tr><td>MB-WNT</td><td>DDX3X</td><td>KMT2D</td><td>co</td><td>cooperation
false positive</td><td>[57]</td></tr>
<tr><td></td><td colspan="5">Cooperation: Synergistic mechanism is unclear, both genes are involved in gene expression regulation [57]. Candidate might be a false positive: only detected with the permutation test, high p- and FDR-values, but both genes are SMGs and known tumor suppressors.</td></tr>
</table>

cancer type	gene1	gene2	co/me	possible explanation	reference(s)
PAN	HLA-A	PCDHA8	co	false positive	
	NCAM1	PCDHA8	co		
	PCDHA8	ZNF721	co		
	**False positives**: At first sight interesting candidates, because all co-occurring mutated gene pairs are found in MB samples (different subgroups), and PCDHA8 is involved in brain development and function. However, all mutations are identical in the three MB samples that show co-occurrence, and these are known common variants. Taken together, these candidates probably resulted from a technical artifact.				
	PIK3CA	TP53	me	pathway epistasis	[58]
	**Pathway epistasis**: We found a PAN-cancer wide mutual exclusivity relationship between TP53 and PIK3CA in the DKFZ data set. While in six cancer types both genes are mutated in a total of 69 samples, in only 2 samples these mutations co-occur. Several previous studies also found mutual exclusive patterns between mutations in the p53 and PI3K pathways and suggested a redundant effect where either event is sufficient for tumor growth.[58]				

TARGET

cancer type	gene1	gene2	co/me	possible explanation	reference(s)
AML	CEBPA	CSF3R	co	cooperation	[59,60]
	**Cooperation**: activating mutations in CSF3R cooperate with loss of function mutations in CEBPA to promote acute leukemia development [59]. Co-occurrence was validated in other study [60].				
	CEBPA	WT1	co	cooperation	[61,62]
	**Cooperation**: AML patients with both CEBPA and WT1 mutations have worse outcome than with CEBPA mutation alone[61]. Mechanism is unknown, but review paper[62] suggests a role of epigenetic regulation, since mutations in genes that disturb DNA methylation (such as WT1) are often found in patients with bi-allelic CEBPA mutations.				
	FLT3	IDH2	co	cooperation	[63–65]
	**Cooperation**: IDH2^{R140Q} cooperates with Flt3^{ITD} (FLT3 in-frame duplications) in leukemia initiation[63]. Both Flt3^{ITD} and FLT3-TKD (point mutations in tyrosine kinase domain) induce activation of the FLT3 receptor, leading to activation of RAS and PI3K signaling pathways [64,65]. The samples in our data set that have co-occurring FLT3 and IDH2 mutations indeed have the IDH2^{R140Q} and FLT3-TKD mutation, suggesting that co-operation was the cause of their co-occurrence.				
	IDH2	NPM1	co	cooperation	[64]
	**Cooperation**: IDH2 and NPM1 mutations cooperate to activate Hoxa9/Meis1 and hypoxia pathways in AML[64].				
	FLT3	KIT	me	pathway epistasis	[66]
	KIT	NRAS	me		
	**Pathway epistasis**: RAS genes are downstream of KIT in the signaling receptor tyrosine kinase (RTK) signaling pathway. The module FLT3-KIT-NRAS was also found with CoMEt, a method that finds modules of mutually exclusive genes in order to detect pathway epistasis [66].				
	NRAS	WT1	me	synthetic lethality	[37,67]
	KIT	WT1	me		
	**Synthetic lethality**: a study shows that WT1 was required in cells expressing oncogenic KRAS but not in cells expressing wild-type KRAS. Inactivation of WT1 inhibited KRAS-dependent tumor formation [37,67]. RAS genes are downstream of KIT in the RTK signaling pathway, so activation of KIT leads to activation of RAS. The synthetic lethal link between KRAS and WT1 (see above) might also apply to genes involved in the RTK/RAS pathway, such as KIT and NRAS.				

cancer type	gene1	gene2	co/me	possible explanation	reference(s)
B-ALL	PXDN	ZNF582	co *	false positive	
	**False positive**: only found with permutation test, in both B-ALL and PAN test, but signal (co-occurrence) comes from 3 B-ALL samples only. None of the genes are SMGs. No literature found on relation between both genes.				
	CRLF2	NRAS	me	subtype	[68]
	**Subtype**: CRLF2 mutations typically occur in Ph-Like B-ALL and IgH@ rearrangements, while RAS mutations are often found in near-haploid B-ALL[56].				
	FLT3	KRAS	me *	pathway epistasis	[66,69,70]
	**Pathway epistasis**: Both genes are involved in the RTK-RAS signaling [66,69.70].				
	KRAS	TP53	me *	subtype	[68]
	**Subtype**: in aneuploid B-ALL, TP53 mutations are found in almost all low hypodiploid (32-39 chromosomes) cases, but not in near haploids; RTK-RAS mutations are specific in near haploid (24-31 chromosomes) cases and are rarely found in low hypodiploids[68].				
T-ALL	JAK1	JAK3	co *	cooperation	[71]
	**Cooperation**: Cooperating JAK1 and JAK3 mutants activate STAT transcription factors and increase resistance to JAK inhibitors[71].<br>**False positive**: genes both have large MLA (mutation load association, see table S3).				
	JAK3	STAT5B	co	cooperation	[72,73]
	**Cooperation**: Mutant JAK3 and STAT5B both play a role in activation of the Jak/Stat signaling pathway and have been shown to co-occur in other studies as well [72]. Furthermore, it has been shown that both genes cooperate with HOXA9 signaling, each in a different manner [73].				
	NRAS	WT1	co	cooperation	[74]
	**Cooperation**: the gene-pair NRAS-WT1 is a mutually exclusive candidate in AML but is co-occurring in T-ALL. WT1 can act both as tumor suppressor and oncogene, depending on cell type, developmental stage or which isoform is expressed[38]. While WT1 was shown to be essential for cells expressing oncogenic KRAS[67] (see NRAS-WT1 in AML), another study showed that WT1 blocked tumor promoting activity of Ras[74]. This suggests that inactivating mutations in WT1 can increase Ras activity.				
	DNM2	USP7	me *	subtype	[31]
	**Subtype**: USP7 is typically mutated in TAL1 subgroup, DNM2 is typically mutated in TLX3 subgroup[31].				
	FBXW7	JAK3	me *	pathway epistasis	[29,75,76]
	**Pathway epistasis**: mutations in FBXW7 activate NOTCH1 pathway resulting downstream in activation of Akt pathway[29,75]. Mutations in JAK3 also activate the Akt pathway.[76]<br>**False positive**: genes have large ΔMLA (difference in mutation load association, see table S3).				
	FBXW7	PTEN	me *	pathway epistasis	[29,75]
	**Pathway epistasis**: Activating mutations in FBXW7 activate NOTCH1 pathway resulting downstream in activation of Akt pathway (via activation of HES1, which represses PTEN)[29]. Inactivation of PTEN will have same result. [75]				
	JAK3	PTEN	me	pathway epistasis<br>subtype<br>false positive	[76]<br>[31]
	**Pathway epistasis**: JAK3 and PTEN regulate downstream pathways RAS-MEK-ERK and PI3K-AKT. [76]<br>**Subtype**: JAK3 is typically mutated in HOXA subgroup, PTEN is typically mutated in TAL1 subgroup[31].<br>**False positive**: genes have large ΔMLA (difference in mutation load association, see table S3).				
	MAGI1	NOTCH1	me	pathway epistasis	[75,77]
	**Pathway epistasis**: Mutations in Notch result in activation of Akt (via activation of HES1, which represses PTEN)[75]. MAGI-1 inhibits Akt activity[77] , so if mutated MAGI1 activates Akt activity it has the same (redundant) effect as NOTCH1 mutations.<br>**False positive**: genes have large ΔMLA (difference in mutation load association, see table S3).				

cancer type	gene1	gene2	co/me	possible explanation	reference(s)
T-ALL (cont.)	NOTCH1	USP7	me	synthetic lethality	[78]

cancer type	gene1	gene2	co/me	possible explanation	reference(s)
				false positive	
	**Synthetic lethality**: USP7 corporates with NOTCH1[78]. The NOTCH1 complex interacts with and is stabilized by USP7. Mutations in USP7 might destabilize the NOTCH1 pathway and could therefore be mutually exclusive with NOTCH1 activating mutations.<br>**False positive**: genes have large ΔMLA (difference in mutation load association, see table S3).				
	PHF6	PIK3R1	me	subtype	30,31
	**Subtype**: PHF6 is typically mutated in TLX3 subgroup, PIK3R1 is typically mutated in TAL1 subgroup[30,31].				
	PHF6	PTEN	me *	subtype<br>false positive	30,31
	**Subtype**: PHF6 is typically mutated in TLX3 subgroup, PTEN is typically mutated in TAL1 subgroup[30,31].<br>**False positive**: genes have large ΔMLA (difference in mutation load association, see table S3).				
	PHF6	USP7	me *	subtype<br>false positive	31
	**Subtype**: USP7 is typically mutated in TAL1 subgroup, PHF6 is typically mutated in TLX3 subgroup[31].<br>**False positive**: genes have large ΔMLA (difference in mutation load association, see table S3).				
	PTEN	WT1	me *	pathway epistasis<br>subtype	30,74,76<br>31
	**Pathway epistasis**: WT1 plays a role in the RAS-MEK-ERK pathway as WT1 transactivates two negative regulators of the Ras/MAPK pathway [74] . PTEN is negatively regulates the PI3K-AKT-mTOR pathway[30]. Loss of function mutations in WT1 and PTEN leads to the activation of each of these signaling pathways. It has been shown that the RAS-MEK-ERK pathway acts as compensatory pathway after inhibition of the PI3K-AKT-mTOR pathway[76]. This suggests that activation of either pathway is enough for tumor progression.<br>**Subtype**: PTEN is typically mutated in TAL1 subgroup, WT1 is typically mutated in TLX3 subgroup[31]. Note however that mutual exclusivity holds within subgroups.				
	USP7	WT1	me	subtype	31
	**Subtype**: USP7 is typically mutated in TAL1 subgroup, WT1 is typically mutated in TLX3 subgroup[31].				
WT	CTNNB1	EFCAB6	co	cooperation	79
	**Cooperation**: both genes are involved in androgen receptor regulation; known case of adult who developed Wilms tumor after using large amounts of anabolic androgenic steroids [79].<br>EFCAB6 mutations might be **false positives**: the 3 patients with EFCAB6 mutations all have the L774Q mutation and 2 of them also have the neighboring mutation L773P.				
	DROSHA	TP53	me	subtype	80
	**Subtype**: the strong enrichment of TP53 mutations in Wilms tumors with Diffuse Anaplasia (DAWT) and of DROSHA mutations in Wilms tumors with Favorable Histology (FHWT) suggests cancer subtype as underlying cause of the ME relationship [80]. However, both mutated genes do occur in both subtypes and sporadically even co-occur in the same patient [80].				
PAN	FLT3	JAK2	me	pathway epistasis<br>subtype	65,81,82<br>56
	**Pathway epistasis**: mutually exclusive in B-ALL (largest group), AML, T-ALL and OS; both genes are tyrosine kinases and their mutants activate downstream proliferative signaling pathways: PI3K-Akt, RAS, Jak-STAT[65,82]. JAK family mutations are associated with resistance to FLT3 inhibitors in AML [81].<br>**Subtype**: in B-ALL, FLT3 mutations are enriched in high-hyperdiploid and MLL arranged subgroups, while JAK2 mutations are frequent in BCR-ABL1 (-like) and CRLF2 mutated subgroups [68].				

*co: co-occurrence; me: mutual exclusivity; *: also significant in PAN cancer test*

Supplementary Figure 1: MLA distribution per TARGET cancer type

Distribution of MLA scores for each TARGET cancer type in which candidate genetic interactions were found. Genes that are part of at least one candidate gene pair are shown with their MLA score and mutation frequency (secondary vertical axis).

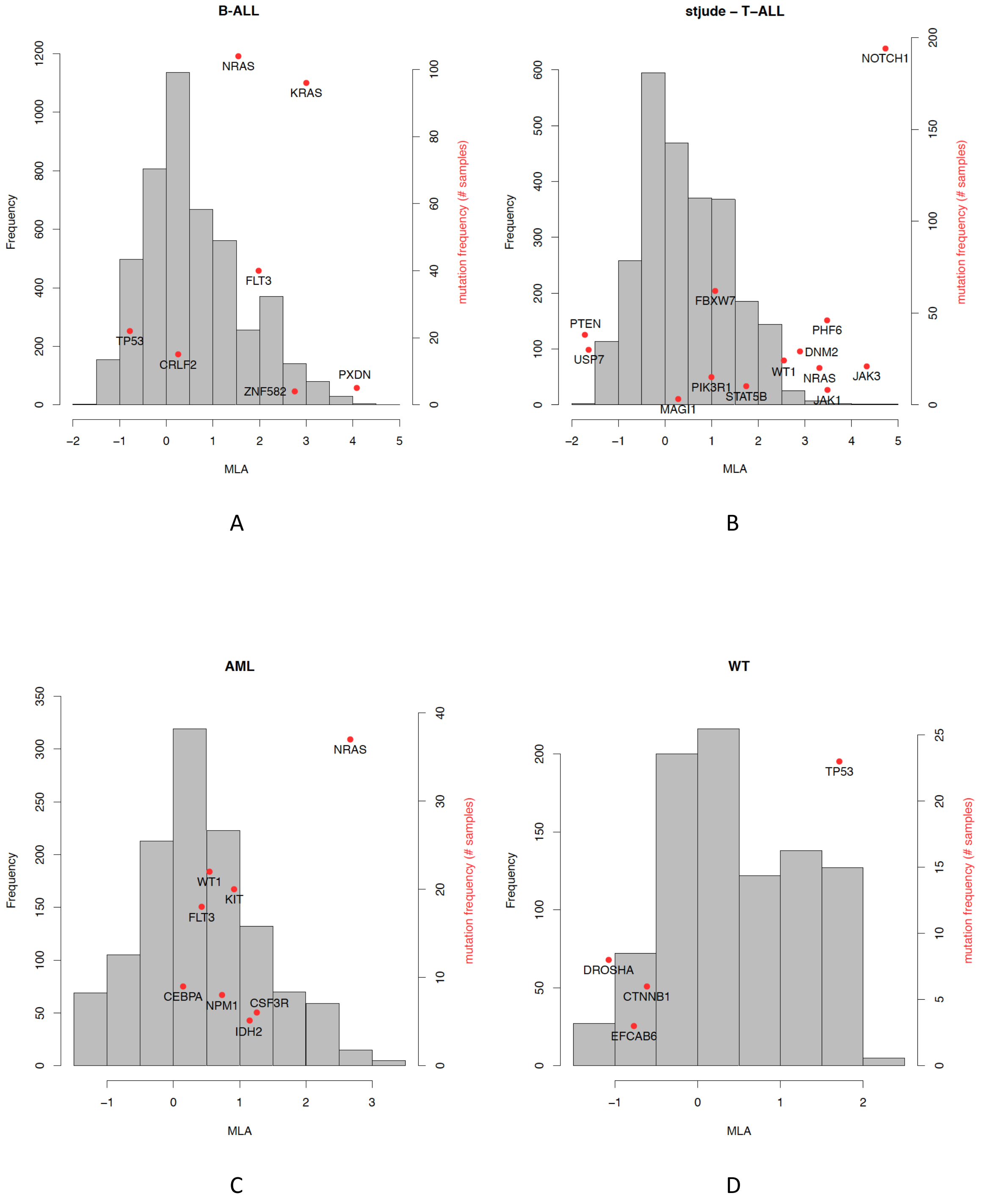

Supplementary Figure 2: MLA distribution per DKFZ cancer type

Distribution of MLA scores for each DKFZ cancer type in which candidate genetic interactions were found. Genes that are part of at least one candidate gene pair are shown with their MLA score and mutation frequency (secondary vertical axis).

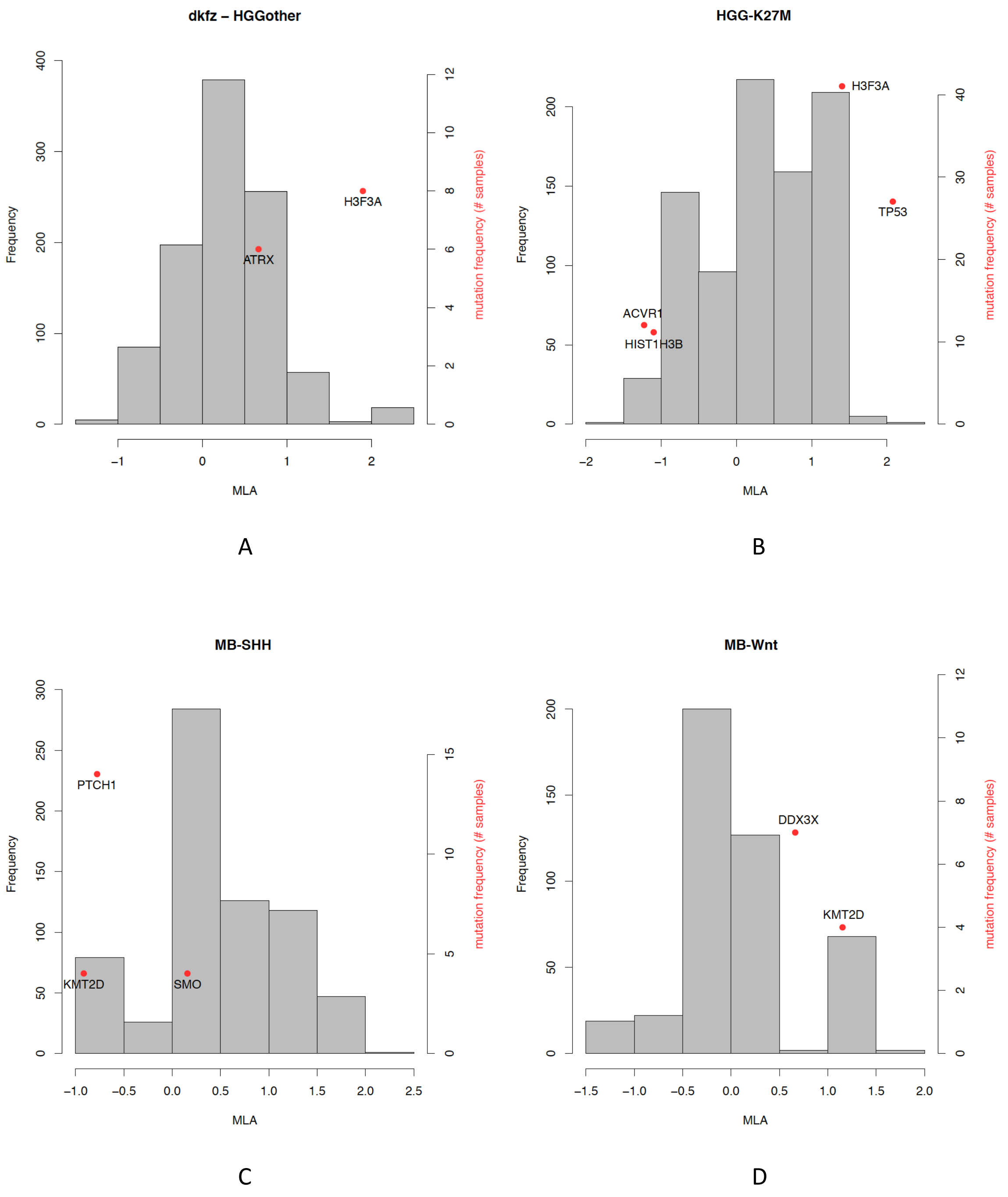

Supplementary Figure 3: Tumor load and mutation frequency of suspect gene pairs

Mutation frequency heatmaps (A-G) of both genes in the seven ‘suspect’ T-ALL candidate gene pairs (having at least one gene with MLA > 3 and having ΔMLA > 3 in case of mutual exclusivity or both genes having an MLA > 3 in co-occurrent pairs). Samples are ordered on tumor mutation load (TML).

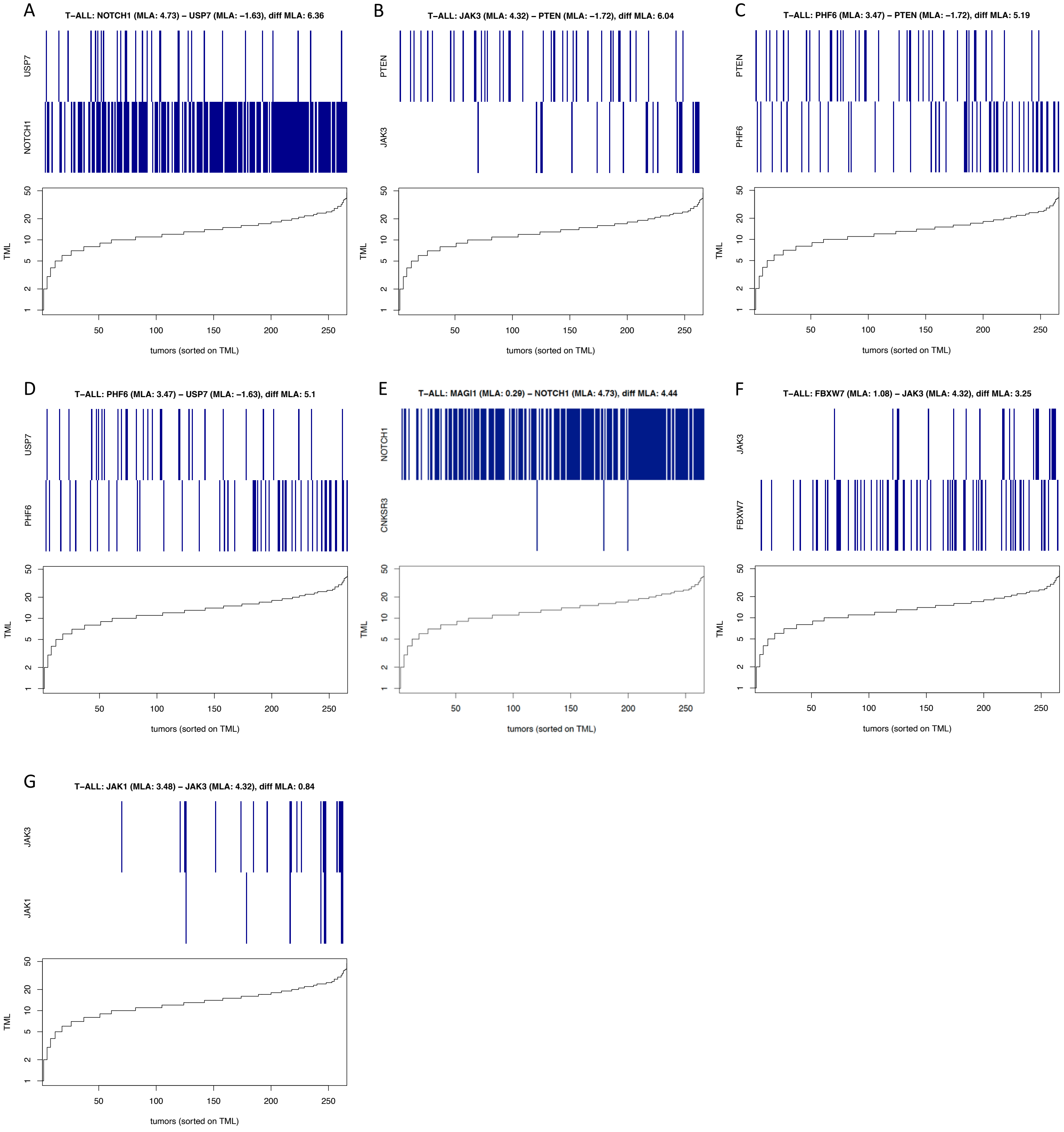

Supplementary Figure 4: Comparison p-values Permutation and WeSME test

P-values resulting from testing gene pairs in all cancer types (excluding the PAN cancer test) with the Permutation test compared to their corresponding p-values produced with the WeSME test. The WeSME test was run ten times, but only the results from the first run are shown for better comparison. Each dot represents a gene pair tested for co-occurrence (CO, blue) or mutual exclusivity (ME, red). Only gene pairs with co-occurrence count > 2 were tested (see Materials and Methods), explaining the small proportion of CO data points.

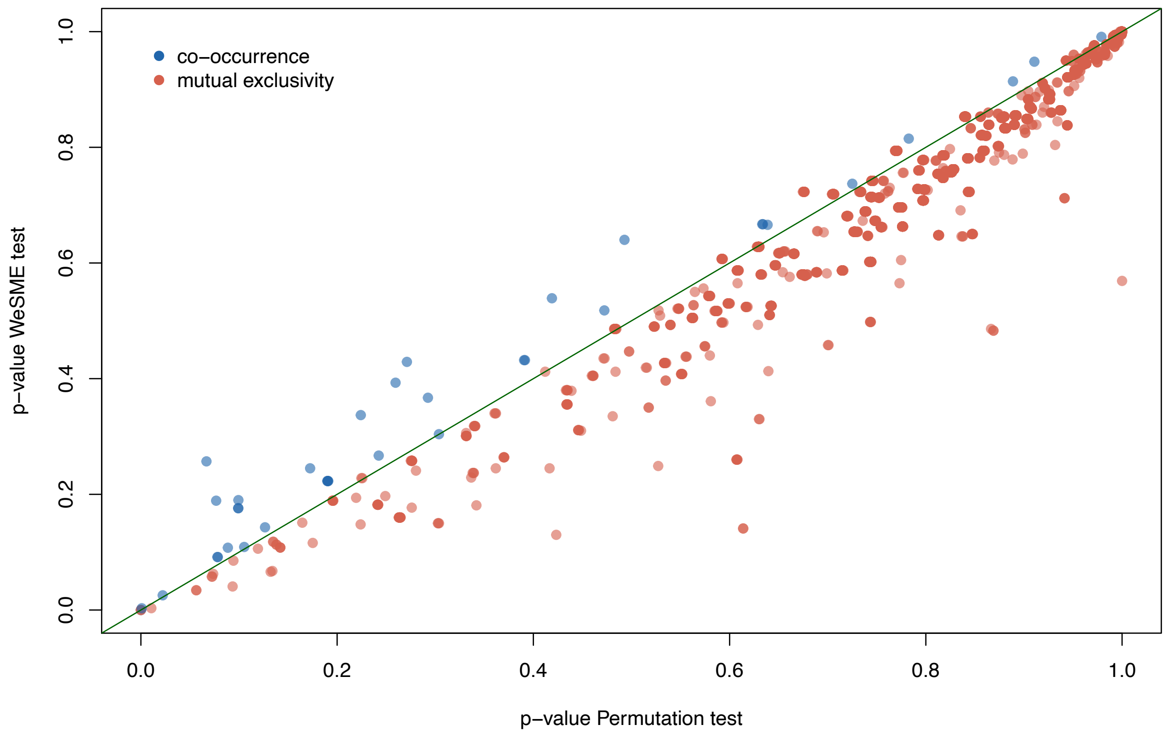

Supplementary Figure 5: Detailed workflow of Genetic Interactions pipeline

Starting with a gene-sample mutation matrix, the permutation test counts the number of co-occurrences for each gene pair and compares this count to a null distribution based on matrix randomizations (*N=1,000,000*) to compute p-values. The same gene-sample matrix serves as input for the WeSME test. This test compares mutual exclusivity counts with a null distribution generated by weighted sampling based on sample-level mutation rates. False discovery rates are estimated by comparing p-values with a null distribution of p-values. This null distribution is created by testing 100 (Permutation test) or 300 (WeSME test) random matrices. This figure is based on Figure 1 in Park and Lehner[23] and Figure 1 in Kim, Madan, and Przytycka[28].

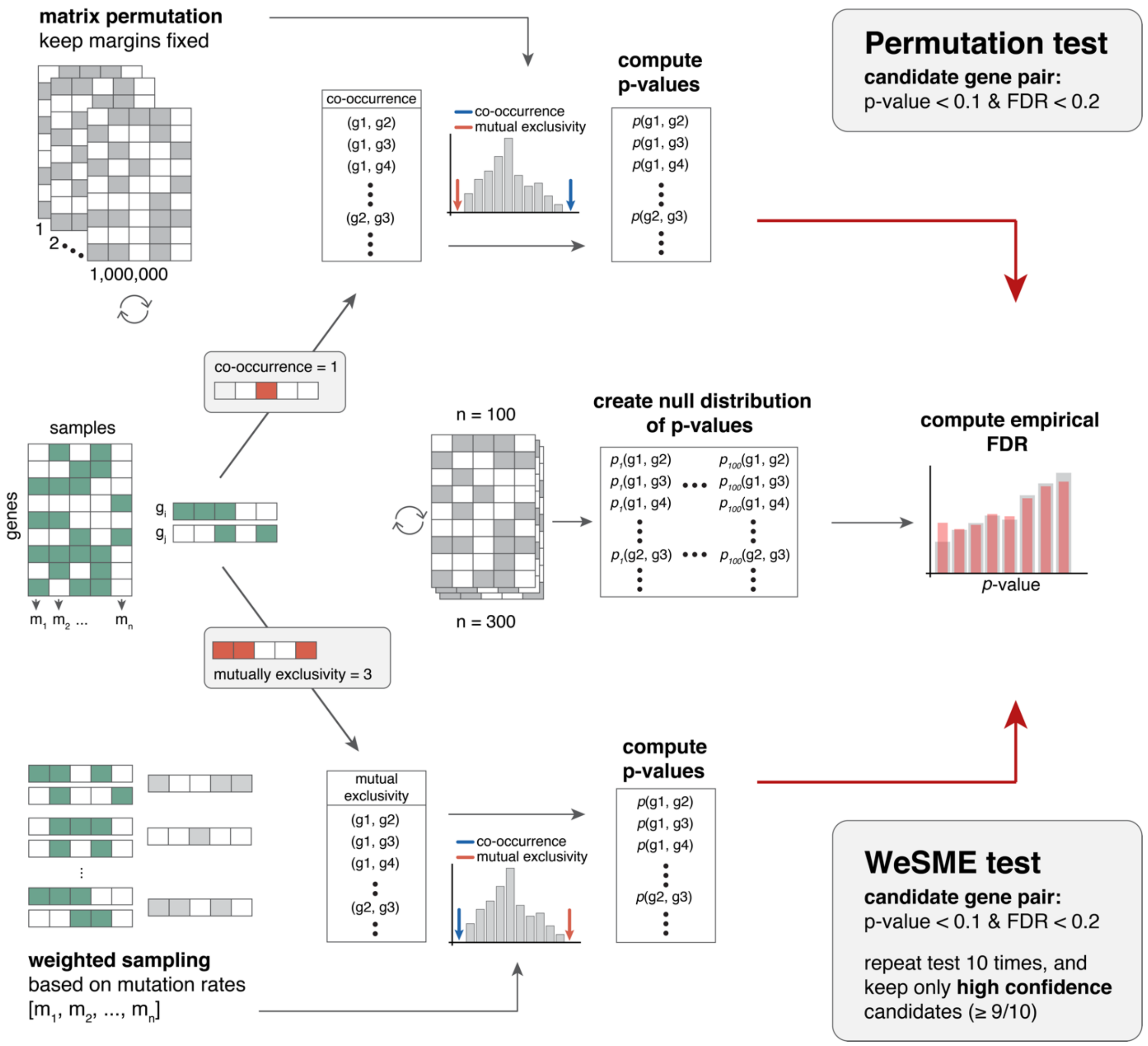

Supplement: Supplementary file 2 — Supplementary Information [file 42003_2021_2647_MOESM2_ESM.pdf]
