# Supplementary material for: A systematic analysis of genetic interactions and their underlying biology in childhood cancer
Source: Commun Biol. 2021 Oct 6;4:1139. doi: 10.1038/s42003-021-02647-4 (PMC8494736; doi:10.1038/s42003-021-02647-4)
Supplement: Supplementary file 3 — Description of Supplementary Files [file 42003_2021_2647_MOESM3_ESM.pdf]

Description of Additional Supplementary Files

File name: Supplementary Data 1

Description: Candidate gene pairs in TARGET and DKFZ data set.

File name: Supplementary Data 2

Description: Source data for Fig. 1, 4 & 5.
